# 2-Ethyl-1*H*-imidazol-3-ium hemioxalate oxalic acid monohydrate

**DOI:** 10.1107/S1600536811020733

**Published:** 2011-06-11

**Authors:** Run-Qiang Zhu

**Affiliations:** a Ordered Matter Science Research Center, College of Chemistry and Chemical Engineering, Southeast University, Nanjing 211189, People’s Republic of China

## Abstract

In the title compound, C_5_H_9_N_2_^+^·0.5C_2_O_4_^2−^·C_2_H_2_O_4_·H_2_O, the anions, cations and water mol­eculars are linked by N—H⋯O and O—H⋯O hydrogen bonds which define a tightly bound three-dimensional structure. The title compound is a layered structure as viewed along the *a* or *c* axis; one layer contains water and oxalic acid mol­ecules, the other the imidazolium cation. The C atoms of the ethyl group of the 2-ethyl­imidazolium cation are disordered over two positions of equal occupancy.

## Related literature

For general background to ferroelectric organic frameworks, see: Fu *et al.* (2009 ▶); Ye *et al.* (2006 ▶); Zhang *et al.* (2008 ▶, 2010 ▶).
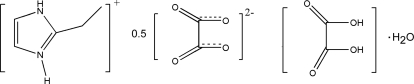


## Experimental

### 

#### Crystal data

C_5_H_9_N_2_^+^·0.5C_2_O_4_^2−^·C_2_H_2_O_4_·H_2_O
*M* *_r_* = 249.20
Monoclinic, 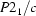

*a* = 6.971 (3) Å
*b* = 15.716 (7) Å
*c* = 10.484 (4) Å
β = 93.736 (8)°
*V* = 1146.1 (8) Å^3^
*Z* = 4
Mo *K*α radiation
μ = 0.13 mm^−1^
*T* = 293 K
0.30 × 0.25 × 0.20 mm

#### Data collection

Rigaku SCXmini diffractometer
Absorption correction: multi-scan (*CrystalClear*; Rigaku, 2005 ▶) *T*_min_ = 0.962, *T*_max_ = 0.975
12371 measured reflections
2614 independent reflections
2192 reflections with *I* > 2σ(*I*)
*R*_int_ = 0.039

#### Refinement

*R*[*F* ^2^ > 2σ(*F* ^2^)] = 0.046
*wR*(*F* ^2^) = 0.115
*S* = 1.08
2614 reflections
183 parameters
3 restraints
H atoms treated by a mixture of independent and constrained refinement
Δρ_max_ = 0.35 e Å^−3^
Δρ_min_ = −0.44 e Å^−3^

### 

Data collection: *CrystalClear* (Rigaku, 2005 ▶); cell refinement: *CrystalClear*; data reduction: *CrystalClear*; program(s) used to solve structure: *SHELXS97* (Sheldrick, 2008 ▶); program(s) used to refine structure: *SHELXL97* (Sheldrick, 2008 ▶); molecular graphics: *SHELXTL* (Sheldrick, 2008 ▶); software used to prepare material for publication: *SHELXL97*.

## Supplementary Material

Crystal structure: contains datablock(s) I, global. DOI: 10.1107/S1600536811020733/qm2009sup1.cif

Structure factors: contains datablock(s) I. DOI: 10.1107/S1600536811020733/qm2009Isup2.hkl

Supplementary material file. DOI: 10.1107/S1600536811020733/qm2009Isup3.cml

Additional supplementary materials:  crystallographic information; 3D view; checkCIF report

## Figures and Tables

**Table 1:** Hydrogen-bond geometry (Å, °)

*D*—H⋯*A*	*D*—H	H⋯*A*	*D*⋯*A*	*D*—H⋯*A*
N1—H1*B*⋯O3^i^	0.86	2.01	2.855 (2)	166
N1—H1*B*⋯O1^i^	0.86	2.53	3.026 (2)	118
N2—H2*A*⋯O7^ii^	0.86	2.17	2.949 (2)	151
N2—H2*A*⋯O3	0.86	2.47	3.060 (2)	126
O1—H1*A*⋯O6^ii^	0.82	1.70	2.4800 (16)	159
O4—H4⋯O7^ii^	0.82	1.71	2.5283 (17)	174
O7—H7*A*⋯O5^ii^	0.84 (3)	1.87 (3)	2.7006 (18)	166 (2)
O7—H7*B*⋯O5^iii^	0.88 (3)	1.82 (3)	2.6856 (19)	169 (2)
O7—H7*B*⋯O6^iv^	0.88 (3)	2.47 (3)	2.923 (2)	113 (2)

Symmetry codes: (i) 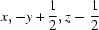; (ii) 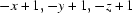; (iii) 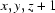; (iv) 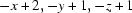.

**Fig. 1.**
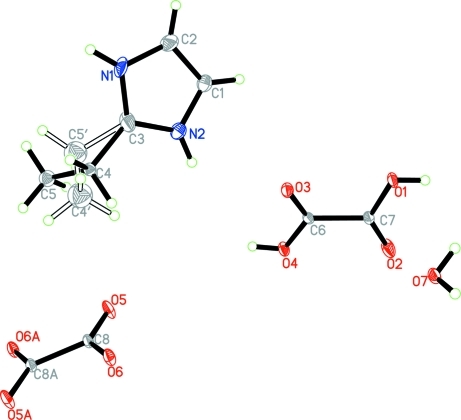
A partial packing diagram of the title compound, with the displacement ellipsoids were drawn at the 30% probability level.

**Fig. 2.**
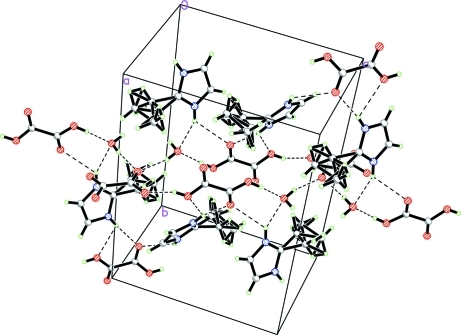
Packing diagram of the title compound, hydrogen bonds are shown as dashed lines.

## supplementary crystallographic information

### Comment

The basic method to find potential ferroelectric phase change material is Dielectric constant measurement of compounds with temperature (Fu *et al.*, 2009; Ye *et al.*, 2006; Zhang *et al.*, 2008; Zhang *et al.*, 2010). Unfortunately, the title compound's dielectric dose not appear strange from 80 K to 298 K (m.p.219–229).

By X-ray diffraction analysis in 123 K, we can define the structure of title compound as Fig.1. Title compound, of the formula [C_5_H_9_N_2_] _2_^+^ [C_2_O_4_]^2-^.C_2_H_2_O_4_.2H_2_O, was obtained by 2 - ethyl imidazole, oxalic acid in water in basic aqueous solution and was isolated as colourless crystals. The whole molecules are organized in a three-dimensional structure involving hydrogen bonds, both intercationic and between cations and water molecules. The O···O distances of the hydrogen bonding are observed to be in the range of 2.476 (3)–2.924 (3) Å and the N···O distances of the hydrogen bonding are in the range of 2.054 (2) - 3.059 (2) Å for table1.

### Experimental

A mixture of 2 - ethyl imidazole (2.4 g, 25 mmol), oxalic acid (3.15 g, 25 mmol) in water was stirred for several days at ambient temperature, Colourless sheet crystals were obtained.

### Refinement

Positional parameter of all the H atoms except for H7A and H7B were calculated geometrically and the H atoms were set to ride on the C atoms to which they are bonded, with *U*_iso_(H) =1.2Ueq(C). The H7A and H7B on the O7 was freely refined.

### Crystal data

**Table:**

C_5_H_9_N_2_^+^·0.5C_2_O_4_^2^^−^·C_2_H_2_O_4_·H_2_O	*Z* = 4
*M**_r_* = 249.20	*F*(000) = 524.0
Monoclinic, *P*2_1_/*c*	*D*_x_ = 1.444 Mg m^−^^3^
Hall symbol: -P 2ybc	Mo *K*α radiation, λ = 0.71073 Å
*a* = 6.971 (3) Å	θ = 2.3–27.5°
*b* = 15.716 (7) Å	µ = 0.13 mm^−^^1^
*c* = 10.484 (4) Å	*T* = 293 K
β = 93.736 (8)°	Prism, colourless
*V* = 1146.1 (8) Å^3^	0.30 × 0.25 × 0.20 mm

### Data collection

**Table:**

Rigaku SCXmini diffractometer	2614 independent reflections
Radiation source: fine-focus sealed tube	2192 reflections with *I* > 2σ(*I*)
graphite	*R*_int_ = 0.039
CCD_Profile_fitting scans	θ_max_ = 27.5°, θ_min_ = 2.9°
Absorption correction: multi-scan (*CrystalClear*; Rigaku, 2005)	*h* = −9→9
*T*_min_ = 0.962, *T*_max_ = 0.975	*k* = −20→20
12371 measured reflections	*l* = −13→13

### Refinement

**Table:**

Refinement on *F*^2^	Secondary atom site location: difference Fourier map
Least-squares matrix: full	Hydrogen site location: inferred from neighbouring sites
*R*[*F*^2^ > 2σ(*F*^2^)] = 0.046	H atoms treated by a mixture of independent and constrained refinement
*wR*(*F*^2^) = 0.115	*w* = 1/[σ^2^(*F*_o_^2^) + (0.0549*P*)^2^ + 0.3631*P*] where *P* = (*F*_o_^2^ + 2*F*_c_^2^)/3
*S* = 1.08	(Δ/σ)_max_ < 0.001
2614 reflections	Δρ_max_ = 0.35 e Å^−^^3^
183 parameters	Δρ_min_ = −0.44 e Å^−^^3^
3 restraints	Extinction correction: *SHELXL97* (Sheldrick, 2008)
Primary atom site location: structure-invariant direct methods	Extinction coefficient: 0

### Special details

**Table:**

Geometry. All e.s.d.'s (except the e.s.d. in the dihedral angle between two l.s. planes) are estimated using the full covariance matrix. The cell e.s.d.'s are taken into account individually in the estimation of e.s.d.'s in distances, angles and torsion angles; correlations between e.s.d.'s in cell parameters are only used when they are defined by crystal symmetry. An approximate (isotropic) treatment of cell e.s.d.'s is used for estimating e.s.d.'s involving l.s. planes.
Refinement. Refinement of *F*^2^ against ALL reflections. The weighted *R*-factor *wR* and goodness of fit *S* are based on *F*^2^, conventional *R*-factors *R* are based on *F*, with *F* set to zero for negative *F*^2^. The threshold expression of *F*^2^ > σ(*F*^2^) is used only for calculating *R*-factors(gt) *etc*. and is not relevant to the choice of reflections for refinement. *R*-factors based on *F*^2^ are statistically about twice as large as those based on *F*, and *R*- factors based on ALL data will be even larger.

### Fractional atomic coordinates and isotropic or equivalent isotropic displacement parameters (Å^2^)

**Table:**

	*x*	*y*	*z*	*U*_iso_*/*U*_eq_	Occ. (<1)
C1	0.1308 (3)	0.28168 (12)	0.2483 (2)	0.0343 (4)	
H1	0.0466	0.3066	0.3025	0.041*	
C2	0.1140 (3)	0.20420 (12)	0.1945 (2)	0.0364 (5)	
H2	0.0158	0.1652	0.2044	0.044*	
C3	0.3798 (4)	0.26258 (12)	0.1326 (2)	0.0475 (6)	
C6	0.2852 (2)	0.51305 (9)	0.44765 (14)	0.0159 (3)	
C7	0.1981 (2)	0.55010 (10)	0.56719 (14)	0.0181 (3)	
C8	0.9398 (2)	0.49850 (10)	0.06011 (14)	0.0169 (3)	
N1	0.2691 (3)	0.19373 (9)	0.12234 (15)	0.0358 (4)	
H1B	0.2914	0.1495	0.0774	0.043*	
N2	0.2952 (2)	0.31678 (9)	0.20807 (16)	0.0349 (4)	
H2A	0.3378	0.3666	0.2286	0.042*	
O1	0.14141 (16)	0.48966 (7)	0.64012 (10)	0.0203 (3)	
H1A	0.0967	0.5104	0.7036	0.031*	
O2	0.1875 (2)	0.62570 (8)	0.58419 (13)	0.0393 (4)	
O3	0.31598 (17)	0.43711 (7)	0.43807 (10)	0.0212 (3)	
O4	0.32172 (16)	0.57027 (7)	0.36275 (10)	0.0201 (3)	
H4	0.3622	0.5469	0.2999	0.030*	
O5	0.76442 (16)	0.51317 (9)	0.04421 (11)	0.0278 (3)	
O6	1.03061 (15)	0.48204 (8)	0.16451 (10)	0.0218 (3)	
O7	0.54960 (17)	0.51036 (7)	0.82209 (11)	0.0202 (3)	
H7A	0.442 (4)	0.5011 (14)	0.852 (2)	0.042 (6)*	
H7B	0.631 (4)	0.5075 (15)	0.890 (3)	0.054 (7)*	
C4	0.5878 (6)	0.2787 (2)	0.0900 (3)	0.0317 (8)	0.655 (6)
H4A	0.6477	0.3251	0.1390	0.038*	0.655 (6)
H4B	0.6659	0.2281	0.1040	0.038*	0.655 (6)
C5	0.5713 (5)	0.3010 (2)	−0.0500 (3)	0.0474 (11)	0.655 (6)
H5A	0.6971	0.3114	−0.0788	0.071*	0.655 (6)
H5B	0.4938	0.3511	−0.0627	0.071*	0.655 (6)
H5C	0.5126	0.2546	−0.0976	0.071*	0.655 (6)
C5'	0.6915 (11)	0.2936 (4)	0.0908 (7)	0.0416 (18)	0.345 (6)
H5'A	0.7855	0.3046	0.0299	0.062*	0.345 (6)
H5'B	0.7294	0.2445	0.1408	0.062*	0.345 (6)
H5'C	0.6822	0.3419	0.1459	0.062*	0.345 (6)
C4'	0.4958 (9)	0.2770 (3)	0.0199 (6)	0.0285 (17)	0.345 (6)
H4'A	0.4507	0.3258	−0.0301	0.034*	0.345 (6)
H4'B	0.4980	0.2272	−0.0347	0.034*	0.345 (6)

### Atomic displacement parameters (Å^2^)

**Table:**

	*U*^11^	*U*^22^	*U*^33^	*U*^12^	*U*^13^	*U*^23^
C1	0.0365 (10)	0.0252 (9)	0.0430 (11)	0.0018 (7)	0.0161 (9)	−0.0093 (8)
C2	0.0340 (10)	0.0249 (9)	0.0510 (12)	0.0005 (8)	0.0089 (9)	−0.0090 (9)
C3	0.0680 (14)	0.0176 (8)	0.0619 (14)	0.0043 (8)	0.0436 (12)	−0.0004 (9)
C6	0.0147 (7)	0.0181 (7)	0.0152 (7)	−0.0011 (5)	0.0025 (5)	0.0011 (6)
C7	0.0186 (7)	0.0197 (8)	0.0167 (7)	0.0002 (6)	0.0054 (6)	−0.0007 (6)
C8	0.0158 (7)	0.0207 (7)	0.0148 (7)	0.0004 (6)	0.0047 (6)	−0.0005 (6)
N1	0.0606 (11)	0.0176 (7)	0.0312 (8)	0.0073 (6)	0.0169 (8)	−0.0054 (6)
N2	0.0422 (9)	0.0179 (7)	0.0466 (10)	0.0011 (6)	0.0166 (8)	−0.0085 (7)
O1	0.0273 (6)	0.0199 (6)	0.0149 (5)	0.0002 (4)	0.0096 (4)	0.0004 (4)
O2	0.0628 (9)	0.0165 (6)	0.0427 (8)	0.0004 (6)	0.0359 (7)	−0.0034 (5)
O3	0.0300 (6)	0.0157 (5)	0.0188 (6)	0.0006 (4)	0.0085 (5)	0.0001 (4)
O4	0.0275 (6)	0.0173 (5)	0.0166 (5)	0.0009 (4)	0.0089 (4)	0.0024 (4)
O5	0.0141 (6)	0.0540 (8)	0.0158 (6)	0.0034 (5)	0.0051 (4)	0.0023 (5)
O6	0.0172 (6)	0.0350 (7)	0.0134 (5)	0.0028 (5)	0.0037 (4)	0.0025 (5)
O7	0.0159 (6)	0.0284 (6)	0.0168 (6)	0.0005 (5)	0.0046 (5)	−0.0002 (5)
C4	0.0362 (17)	0.0220 (15)	0.0368 (16)	0.0076 (14)	0.0020 (19)	−0.0043 (12)
C5	0.051 (2)	0.048 (2)	0.0449 (17)	0.0109 (16)	0.0211 (17)	0.0148 (16)
C5'	0.028 (4)	0.037 (4)	0.059 (4)	−0.001 (3)	−0.001 (3)	−0.014 (3)
C4'	0.036 (3)	0.023 (3)	0.028 (4)	0.002 (2)	0.010 (3)	−0.002 (2)

### Geometric parameters (Å, °)

**Table:**

C1—C2	1.344 (3)	N2—H2A	0.8600
C1—N2	1.363 (3)	O1—H1A	0.8200
C1—H1	0.9300	O4—H4	0.8200
C2—N1	1.369 (2)	O7—H7A	0.84 (3)
C2—H2	0.9300	O7—H7B	0.88 (3)
C3—N1	1.329 (3)	C4—C5	1.505 (5)
C3—N2	1.327 (2)	C4—H4A	0.9700
C3—C4'	1.491 (6)	C4—H4B	0.9700
C3—C4	1.565 (5)	C5—H5A	0.9600
C6—O3	1.2179 (19)	C5—H5B	0.9600
C6—O4	1.3022 (18)	C5—H5C	0.9600
C6—C7	1.542 (2)	C5'—C4'	1.532 (10)
C7—O2	1.204 (2)	C5'—H5'A	0.9600
C7—O1	1.2970 (19)	C5'—H5'B	0.9600
C8—O5	1.2449 (19)	C5'—H5'C	0.9600
C8—O6	1.2550 (19)	C4'—H4'A	0.9700
C8—C8^i^	1.559 (3)	C4'—H4'B	0.9700
N1—H1B	0.8600		
			
C2—C1—N2	106.81 (16)	C7—O1—H1A	109.5
C2—C1—H1	126.6	C6—O4—H4	109.5
N2—C1—H1	126.6	H7A—O7—H7B	104 (2)
C1—C2—N1	106.87 (17)	C5—C4—C3	107.6 (3)
C1—C2—H2	126.6	C5—C4—H4A	110.2
N1—C2—H2	126.6	C3—C4—H4A	110.2
N1—C3—N2	106.99 (18)	C5—C4—H4B	110.2
N1—C3—C4'	113.7 (3)	C3—C4—H4B	110.2
N2—C3—C4'	131.3 (3)	H4A—C4—H4B	108.5
N1—C3—C4	130.74 (19)	C4—C5—H5A	109.5
N2—C3—C4	121.5 (2)	C4—C5—H5B	109.5
C4'—C3—C4	35.9 (3)	H5A—C5—H5B	109.5
O3—C6—O4	125.24 (14)	C4—C5—H5C	109.5
O3—C6—C7	121.20 (13)	H5A—C5—H5C	109.5
O4—C6—C7	113.55 (13)	H5B—C5—H5C	109.5
O2—C7—O1	127.68 (14)	C4'—C5'—H5'A	109.5
O2—C7—C6	121.60 (14)	C4'—C5'—H5'B	109.5
O1—C7—C6	110.71 (13)	H5'A—C5'—H5'B	109.5
O5—C8—O6	126.16 (14)	C4'—C5'—H5'C	109.5
O5—C8—C8^i^	117.49 (17)	H5'A—C5'—H5'C	109.5
O6—C8—C8^i^	116.35 (16)	H5'B—C5'—H5'C	109.5
C3—N1—C2	109.44 (15)	C3—C4'—C5'	98.8 (5)
C3—N1—H1B	125.3	C3—C4'—H4'A	112.0
C2—N1—H1B	125.3	C5'—C4'—H4'A	112.0
C3—N2—C1	109.87 (16)	C3—C4'—H4'B	112.0
C3—N2—H2A	125.1	C5'—C4'—H4'B	112.0
C1—N2—H2A	125.1	H4'A—C4'—H4'B	109.7

Symmetry codes: (i) −*x*+2, −*y*+1, −*z*.

### Hydrogen-bond geometry (Å, °)

**Table:**

*D*—H···*A*	*D*—H	H···*A*	*D*···*A*	*D*—H···*A*
N1—H1B···O3^ii^	0.86	2.01	2.855 (2)	166.
N1—H1B···O1^ii^	0.86	2.53	3.026 (2)	118.
N2—H2A···O7^iii^	0.86	2.17	2.949 (2)	151.
N2—H2A···O3	0.86	2.47	3.060 (2)	126.
O1—H1A···O6^iii^	0.82	1.70	2.4800 (16)	159.
O4—H4···O7^iii^	0.82	1.71	2.5283 (17)	174.
O7—H7A···O5^iii^	0.84 (3)	1.87 (3)	2.7006 (18)	166 (2)
O7—H7B···O5^iv^	0.88 (3)	1.82 (3)	2.6856 (19)	169 (2)
O7—H7B···O6^v^	0.88 (3)	2.47 (3)	2.923 (2)	113 (2)

Symmetry codes: (ii) *x*, −*y*+1/2, *z*−1/2; (iii) −*x*+1, −*y*+1, −*z*+1; (iv) *x*, *y*, *z*+1; (v) −*x*+2, −*y*+1, −*z*+1.
